# Supplementary material for: Safeguarding in adolescent mental health research: navigating dilemmas and developing procedures
Source: BMJ Open. 2024 Feb 29;14(2):e076700. doi: 10.1136/bmjopen-2023-076700 (PMC10910574; doi:10.1136/bmjopen-2023-076700)
Supplement: Supplementary data [file bmjopen-2023-076700supp001.pdf]

Supplementary Material 1 – Application and justification of SMART Schools Study Safeguarding Procedures

Supplementary Table. SMART Schools Study Safeguarding Procedures and Justification

	Procedures	Justification
1.Pre-Visit	Inform School Staff of Safeguarding Procedure: During recruitment meetings, verbally inform school staff about the safeguarding approach, including an overview of Question 9 PHQ-9.	School staff understand the school and research team roles and responsibilities in safeguarding adolescent mental health, including when they are required to act on information.
	Engage with School DSL: Email the school DSL 7 days before data collection to outline the safeguarding procedures.	The school DSL is the staff member with overarching responsibility and training for pupil mental health. They will have access to information on pupil mental health needs.
	Provide School with Mental Health Resources: Share the SMART Schools Study Mental Health Resource Pack, which provides links to mental health resources, support for pupils, and GP referral services.	To ensure schools are provided with evidence-based resources to support pupil mental health.
	Pupil and Parent/Carer Understanding: Pupils and parents are provided with written information, via pupil and parent/carer information leaflets and flyers, that any safeguarding concerns will be reported to the school DSL.	To provide pupils and parents with written information on confidentiality and safeguarding to maximise understanding.
2. During Data Collection	Timing of Data Collection: Data collection will only take place Monday to Thursday.	To ensure the school DSL has sufficient time to act on information provided.
	Pupil Understanding: Research team verbally inform pupils on the day of data collection that any safeguarding concerns will be reported to the school DSL.	To provide verbal information on confidentiality and safeguarding to maximise pupil understanding.
	Verbally Notify DSL of any Safeguarding Concerns: The DSL (or relevant staff member) is verbally notified in the school context of the number of responses to Question 9 PHQ-9 by the research team.	To ensure the DSL has immediate information on the number of safeguarding alerts, to prepare them sufficiently for intervention/ providing psychological support to pupils.
3. Same day as data collection	Email DSL the Safeguarding Concerns: An email is sent to the DSL outlining the safeguarding details, which includes an explanation of Question 9 on PHQ-9, and a secure and encrypted file transfer of pupil names and their response to PHQ-9 Question 9 on the 4-point Likert scale.	This allows for pupil details to be securely transferred to the DSL and meet GDPR* guidance.
4. Within 24 hours of Data Collection	DSL Receipt of Safeguarding Concerns: The research team asks for confirmation from the DSL of receipt of the safeguarding concern email. A follow up phone call is made if no response is received from DSL within 24 hours.	To ensure the DSL has received the details and is responding according to their school's safeguarding and/or welfare policy.

Abbreviations: PHQ-9 - Patient Health Questionnaire-9; DSL - Designated Safeguarding Lead; GDPR - General Data Protection Regulations
